# Evaluation of Transplant Suitability in a Patient with Previous Colorectal Cancer and Subsequent Radiation Cystitis: Insights from a Complicated Case

**DOI:** 10.1155/2023/7839441

**Published:** 2023-09-25

**Authors:** David Dogahe, Edouard Cubilier, Maxime Taghavi, Saleh Kaysi, Joëlle Nortier, Maria do Carmo Filomena Mesquita

**Affiliations:** Nephrology and Dialysis Department, Brugmann University Hospital, Université Libre de Bruxelles (ULB), Brussels, Belgium

## Abstract

Assessing transplant suitability can be a meticulous process, involving multiple investigations and various specialties. This process is well described in the latest KDIGO guidelines. We recently asked ourselves if those guidelines are still relevant to current clinical practice given the rapid evolution of modern medicine, especially in the field of oncology. We present the complicated case of a 60-year-old woman with ESKD (end-stage kidney disease) and a prior history of cancer, with secondary urological complications, to illustrate different interesting considerations for KT (kidney transplant). Our patient was diagnosed with rectal cancer at the age of 46, for which she was treated with surgery and radiotherapy before developing chronic radiation cystitis. This was followed by repeated urinary tract infections and secondary nephrolithiasis, ultimately leading to severe bilateral hydronephrosis and obstructive ESKD. We know that the type of cancer and its characteristics should be evaluated in detail, and we should offer patient-tailored recommendations after a multidisciplinary evaluation. In our case, the prior rectal cancer is not to be feared because curative treatment has been achieved and the patient has been cancer-free for 14 years, knowing that this type of cancer is not at high risk of recurrence after transplantation. The frail urological anatomy, however, represents a bigger challenge. Not only does it complicate the technical feasibility of KT but it also increases the risk of complications and graft failure. It is difficult to clearly determine KT possibility when considering it in such patients. What is clear on the other hand is that such a decision should be taken considering the choice of the patient and the involved physicians. We should also consider the potential benefits and risks of KT in order to make an informed decision.

## 1. Introduction

Kidney transplantation (KT) offers significant survival and quality of life benefits for patients with end-stage kidney disease (ESKD) when compared to dialysis. It is, therefore, essential to correctly identify and evaluate transplantation possibilities in these patients.

Colorectal cancer is one of the most frequent cancer in Europe, and recent progress in diagnostic evaluation methods and strategies, as well as treatment techniques, has steadily decreased its mortality [1].

Traditionally, KT was feared in patients with a medical history of cancer, but recent advances in cancer therapies and reassuring follow-up studies support a more individualized approach [2, 3] rather than broad guidelines [4] for transplantation suitability assessments.

Urological complications associated with pelvic radiotherapy result from the fact that the bladder, urethra, and distal ureters are in the field of radiation. These complications can be increased depending on the characteristics of radiation therapy. Indeed, older techniques lacked precision, resulting in a higher radiation dose to the surrounding healthy tissue [5]. The urinary tract is particularly sensitive to radiation because of the low proliferative rate of smooth muscle cells, and the adverse effects can develop over a longer time period. Lower urinary tract dysfunction (LUTD) that develops over the years after treatment is linked with bladder fibrosis with increased vesical filling pressures usually leading to renal repercussions [5].

Radiation-induced fistulas are also a feared complication of pelvic radiotherapy. They can also be linked to tissue fibrosis and decreased compliance, leading to high filling pressures that can further damage the tissue, resulting in rupture and fistula formation. New therapies and strategies developed over the years for these complications [1, 5] also justify a revision of KT enlistment limitations for cancer patients in remission.

To illustrate this reflection, we present the KT enlistment challenges we faced in a hemodialyzed patient with colorectal cancer (CRC) in remission.

## 2. Case Presentation

We present the case of a 60-year-old Asian woman followed in our dialysis unit since February 2022 for a severe obstructive renal disease due to chronic radiation cystitis and bilateral hydronephrosis.

Her medical history began in February 2009 with the occurrence of a circumferential rectal adenovillous carcinoma adjacent to the vagina, located two to three centimeters (cm) past the anal margin, staged ypT3N2M0 according to the International Union against Cancer (UICC). The baseline oncological biomarkers were elevated with CEA 11.4 *µ*g/L (NV < 5.2 *µ*g/L) and Ca 19−9 40 kU/L (NV < 27 kU/L).

From May to June 2009, she received a neo-adjuvant radio-chemotherapy composed of 225 mg/m^2^ per week of 5-fluorouracile (5-FU) combined with a total of 45 Gy (18 MV photons in the linear accelerator) rectal radiotherapy split into 25 sessions over 36 days.

On August 2009, she underwent tumor resection by anterior rectal resection at one cm from the anal margin, direct mechanical anocolic anastomosis with the left colon, a protective ileostomy, and a suprapubic catheter.

Pathological analysis revealed a moderately differentiated adenocarcinoma with focal muscular disruptions, respect of the serosa, free resection margins, and tumoral involvement of 11 lymph nodes with capsule disruption, leading to the final UICC grade ypT3N2Mx.

In September 2009, she presented with a postoperative neurogenic bladder and rectovaginal fistula. She also presented with a fecal incontinence and urgencies related to a hypotonic anal sphincter combined with a small volume but functional neo-rectum and a persistent but uncomplicated rectovaginal fistula. At this moment, laboratory results as well as colonoscopy and the computed tomography (CT) scanner were unremarkable. Adjuvant chemotherapy as well as bowel continuity restoration surgery were, therefore, postponed.

The surgical procedure was achieved one year later in October 2010, but it has been decided to pursue follow-up every six months without the adjuvant chemotherapy initially proposed. Positron emission tomography/computed tomography (PET/CT) imaging showed benign reactional abdominal lymph nodes with an inflammatory component of the known fistula.

The subsequent follow-up involving laboratory tests, thoraco-abdominal CT scans, and colonoscopies showed no sign of cancer recurrence up to this day.

In March 2018, an anorectal manometry conducted for severe fecal incontinence resulted in an ischemic perforation of the colon with a pelvic abscess, requiring a permanent colostomy. On March 2022, she underwent surgical treatment for an uncomplicated parastomal eventration.

**Figure 1:**
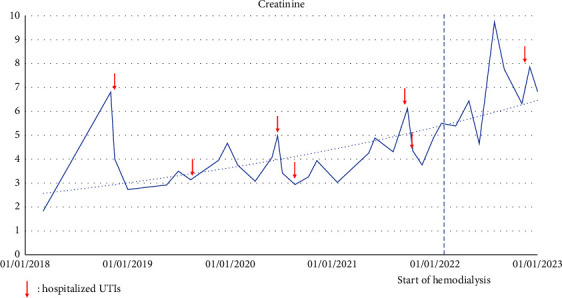
Evolution of creatinine between 2018–2023. Arrow = hospitalized UTIs.

**Figure 2:**
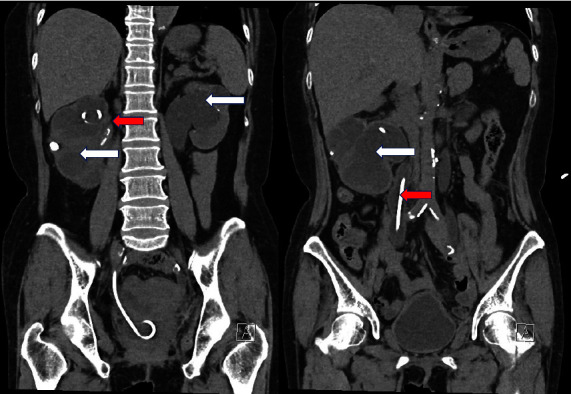
Urinary computed tomography scan showing bilateral ureterohydronephrosis (white arrow) with right double J stent (red arrows).

In parallel, the dysuria secondary to the postoperative neurogenic bladder and chronic radiation cystitis was treated by urethral self-catheterizations, and she presented recurrent urinary tract infections (UTI). At this moment, kidney function was normal. In September 2013, a CT scan revealed a right ureterohydronephrosis with an obstructive urolithiasis in the right ureter which was treated by a double J stent placement. After this episode, multiple UTIs and nephrolithiasis occurred, leading to a progressive concurrent decline of renal function despite a permanent indwelling catheter and a second bilateral nephrostomy in November 2018, as illustrated in Figure 1. She then continued urethral self-catheterizations. To this day, the patient still has severe bilateral hydronephrosis despite a well-placed right double J stent, as illustrated in Figure 2 (Uro-CT).

The renal function inevitably precipitated ESKD and required hemodialysis in February 2022. Unfortunately, the patient has no possible living donor and was considered for listing on cadaveric KT list.

Due to the permanent colostomy, connecting the donor's ureter to the patient's digestive tract is not possible. Also, implanting the donor's ureter in the severely remodeled patient's bladder due to postradiation cystitis seems not feasible. No urodynamic testing was conducted for fear of complications such as fistulae and infection. The pretransplant urological assessment proposed KT from cadaveric donor and permanent ureterostomy; nevertheless, the feasibility of this intervention is still under discussion. Meanwhile, the patient accepted the intervention, well aware of the infectious and oncological risks associated with KT, as she considered the gain in quality of life free from the time-consuming hemodialysis to be greater than the risks.

## 3. Discussion

The latest European Society for Medical Oncology (ESMO) guidelines [1] describes CRC as the most prevalent cancer in Europe, representing 13.6% of all diagnosed cancers, and being the second cause of mortality with 12.2% of all deaths. Privitera et al. [6] report conflicting evidence on the incidence of CRC in KT patients, which seems to be not significantly different from the general population.

However, with respect to the prognosis, each type of cancer should be considered individually by taking into account the presence and characteristics of metastasis and the response to treatment, as well as molecular and genetic features. The TNM staging and treatment options for CRC have changed over the years but usually include a combination of radio-chemotherapy and surgery.

At the time of diagnosis in 2009, our patient was staged ypT3N2M0 according to the UICC. She was scheduled for neo-adjuvant radio-chemotherapy associated with surgery and adjuvant chemotherapy. The latter was not administered due to active postoperative fistulae. Because the cancer remained in remission in follow up, she never pursued the initially programmed adjuvant chemotherapy. The latest KDIGO Clinical Practice Guideline on the Evaluation and Management of Candidates for Kidney Transplantation [4] suggests cancer-specific delays between curative treatments and KT, but these recommendations are based on very low-quality evidence (2D) and require more studies, more so in light of recent and upcoming cancer therapies or genetic diagnostic techniques [2, 3]. The KDIGO guidelines recommended a minimum waiting period between colorectal cancer remission and transplantation as follows: a least 2 years for Dukes A/B, 2 to 5 years for Duke C, and at least 5 years for Duke D. Excluding patients from KT lists based solely on a previous malignancy, especially if confirmed in remission, seems unwise.

Moreover, creating recommendations for KT delays after reaching remission in each type of cancer is a troublesome endeavor given the multiple cancer types, subtypes, therapies, and therapy responsiveness. This level of detail in the consideration of each type of cancer is, therefore, not yet reflected in the current guidelines because of a lack of evidence.

We know that KT recipients are at increased risk of recurrence for certain types of cancer, especially Kaposi sarcoma, nonmelanoma skin cancers, and posttransplant lymphoproliferative disorders. The increased risk in those cancers can be attributed to several factors, amongst which immunosuppression and viral infections play an important role [7]. Management of cancer recurrence or de novo cancer in KT patients is a challenging task. Experts consensus exists on this topic [7] but there is once again a lack of good-quality evidence to support the creation of reliable guidelines on this subject.

However, not all types of cancer are at increased risk of recurrence. Indeed, colorectal cancer confers a lesser risk of recurrence compared to other solid organ cancer, and some data showed no association between waiting time and all-cause mortality after kidney transplantation for those with prior cancer [8].

Our patient presents 14 years of CRC remission state, reassuring recent follow-up, which appears safe for KT enlistment. Also, KT does not seem to promote CRC recurrence, as reported by Chapman et al. [9].

Therefore, CRC in our patient appears to be tangential to difficulties regarding kidney transplantation.

A recent review on urological complications due to radiation therapy by Chorbinska et al. [10] reveals that ureteral stricture and chronic radiation cystitis are both rare complications of pelvic radiotherapy. Treatment options include urinary catheters and nephrostomies, vesical instillations, and other surgical techniques. Despite the multiple urological interventions, our patient progressed into end-stage obstructive renal disease and hemodialysis.

Abnormal bladder is frequent in KT candidate and may represent up to 15% of patients which is a great surgical challenge when considering KT [11]. Initial bladder assessment with urodynamic testing is important for patients with suspected bladder or lower urinary tract dysfunction, as capacities below 100 ml or voiding pressures over 100 cm H20 predispose to transplant complications [11]. Urodynamic testing is, therefore, an important step to guide our transplant options and if pretransplant intervention on the bladder is indicated. Our patient's bladder was considered too fragile due to postradiation modifications of the bladder walls. Techniques using the native bladder were, therefore, ruled out from the start by the urological team, and urodynamic testing was judged to be unnecessary. However, abnormal bladders are not always incompatible with KT when considering the multiple treatment options, such as cystoplasty, ileal conduit formation, continent reservoir use, clean intermittent self-catheterization and cutaneo-ureterostomy.

The opinion of our transplant urological surgeons favored cutaneous ureterostomy as the best option for our patient given the poor quality of the existing bladder tissue. Using intestinal tissue to create a new urinary bladder, such as ileal conduit formation, was also ruled out because of the several previous abdominal surgeries and postradiation abdominal lesions which would make such a technique too difficult in our patient's case. Both techniques are comparable in terms of outcomes, and the choice of technique should be based on the patient's characteristics [10–13]. The simpler surgical technique for cutaneous ureterostomy can make it the preferred technique in frail patients as it is less invasive [10–13]. Some authors [11, 12] have compared KT transplants in patients with urinary diversions and reconstructed bladders and have found that KT is a safe and feasible option in those patients. Their long-term graft and patient survival could be comparable to the general transplant population, depending on the technique used. Transplant in cutaneous ureterostomy seems feasible but is associated with slightly inferior long-term graft and patient survival (respectively, 67% and 78% compared with 83% and 90% in the general KT population) [12].

To the best of our knowledge, there is no other published literature of cases who share the same medical characteristics as our patient. Predicting the outcomes of this KT procedure in our case is, therefore, complicated, as there is little to no evidence to base our opinions upon, which is one of the reasons we chose to share our case.

The case of our patient was discussed with a multidisciplinary expert team consisting of urologists and nephrologists, as well as transplant nephrologists, oncologists, and surgeons, all with experience in KT in patients with prior cancer. These discussions concluded that our patient was at high risk of surgical complications and infections related to the ureterostomy.

This was explained in detail to the patient who still wishes and hopes to be transplanted in order to enjoy a better quality of life.

## 4. Conclusions

With aging populations, the occurrence of patients with prior cancers being considered for transplant will become more frequent. Risk evaluation for recurrence is of certain importance, and new data suggest that colorectal cancer is at a lesser risk. However, risk evaluation can be cumbersome because of the quick evolution of oncology and its therapeutics, allowing better treatment options with fewer complications. This explains that medical literature and guidelines are always one step behind current knowledge. One can hypothesize that the risk of recurrence in KT with a prior history of malignancy may decrease in the future. The guidelines should, therefore, also evolve to reflect the dynamic nature of this field and prevent unnecessary exclusion from a life-improving treatment. It is important to have a multidisciplinary individualized approach in determining which patients with a history of malignancy should be considered for KT.

## Data Availability

The clinical data for this case are stored in hospital medical records and can be accessed upon reasonable request to the corresponding author.

## Abbreviations

ESKD: End-stage kidney disease
KT: Kidney transplant
LUTS: Lower urinary tract dysfunction
CRC: Colorectal cancer
UICC: International Union Against Cancer
5-FU: 5-fluorouracile
CT: Computed tomography
UTI: Urinary tract infection
ESMO: European Society for Medical Oncology.
